# Supplementary figures and images for: Learning spatiotemporal signals using a recurrent spiking network that discretizes time
Source: PLoS Comput Biol. 2020 Jan 21;16(1):e1007606. doi: 10.1371/journal.pcbi.1007606 (PMC7028299; doi:10.1371/journal.pcbi.1007606)

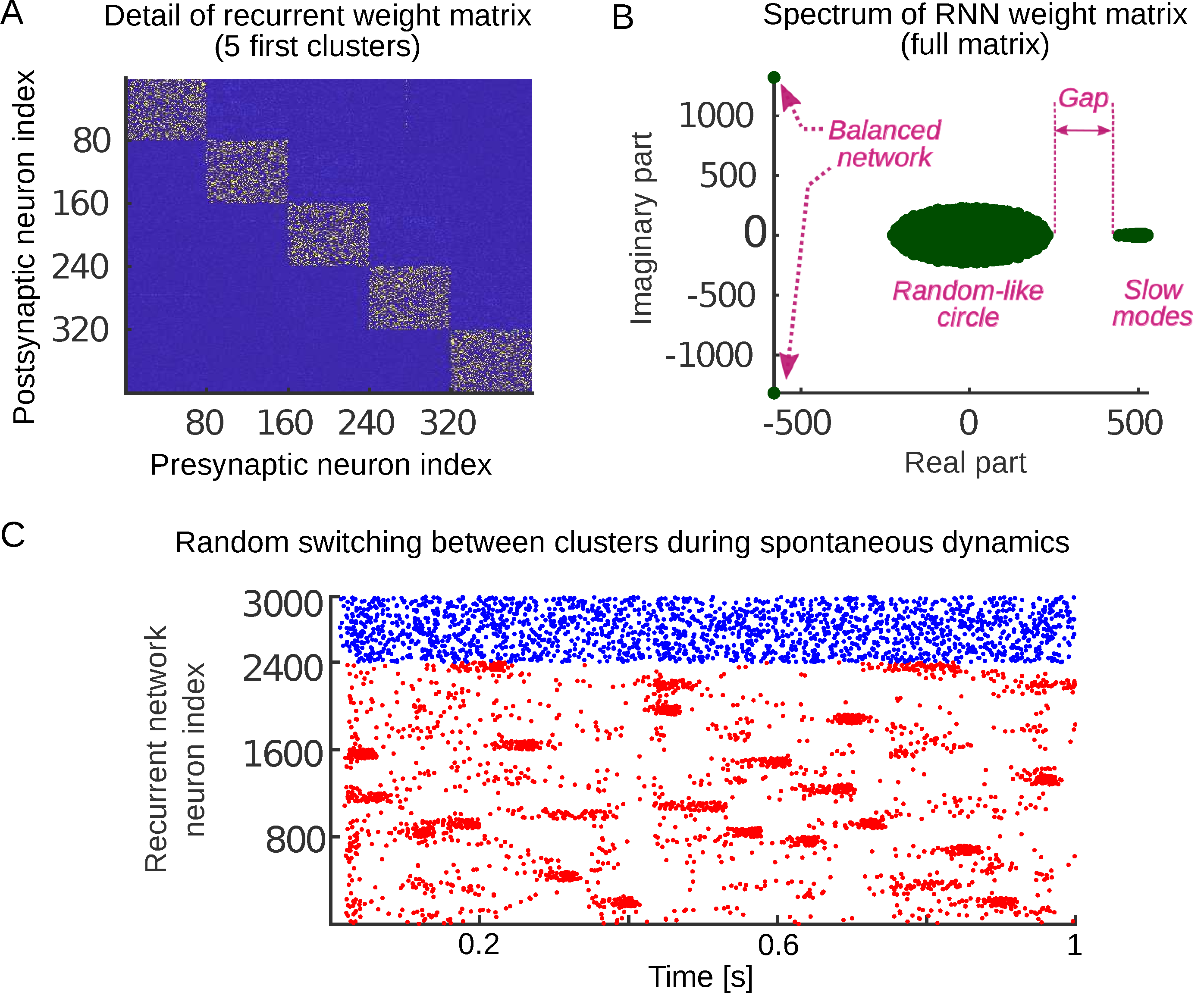

Supplement: S1 Fig — The recurrent network is stimulated with external input that is spatially clustered, but temporally uncorrelated. Each cluster is stimulated for 50 ms, with 50 ms gaps in between stimulations. The rate of external stimulation is 18 kHz during training. This is repeated for 20 minutes after which the network stabilizes during 20 minutes of spontaneous activity. (A) A diagonal structure is learned in the recurrent weight matrix. Since there are no temporal correlations in the external input, there is no off-diagonal structure. (B) The spectrum shows an eigenvalue gap. This indicates the emergence of a slower time scale. The leading eigenvalues do not have an imaginary part, pointing at the absence of feedforward structure and thus there is no sequential dynamics. (C) Under a regime of spontaneous dynamics (i.e. uncorrelated Poisson inputs), the clusters are randomly reactivated. (TIF) [file pcbi.1007606.s001.tif]

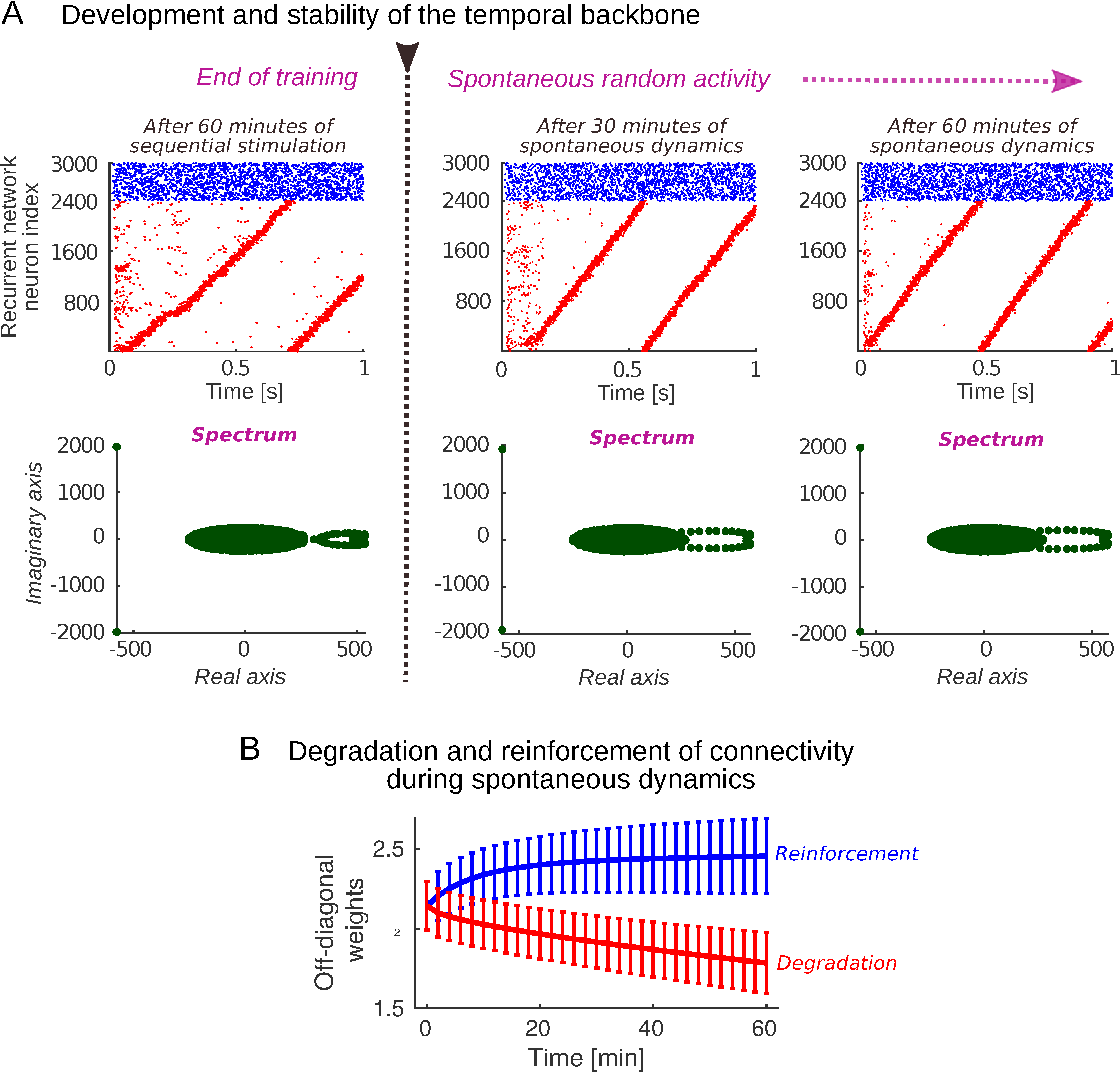

Supplement: S2 Fig — (A) After 60 minutes of training, the network stabilizes during spontaneous activity. During the first 30 minutes of spontaneous dynamics, the connectivity still changes. More specifically, the imaginary parts of the leading eigenvalues increase. This leads to a higher switching frequency and as such a smaller period in the sequential activity. After around 30 minutes, a fixed point is reached. The first row shows spike trains at different times, for one second of spontaneous activity. The second row shows the spectra of the weight matrix at those times. (B) After 60 minutes of sequential stimulation, we test reinforcement and degradation of the learned connectivity by decoupling the plasticity from the dynamics. We plot the evolution of the off-diagonal weights during spontaneous dynamics in two separate cases: (i) we run the dynamics of the network using a frozen copy of the learned weight matrix and apply plastic changes that result from the dynamics to the original weight matrix (blue curve); (ii) we run the dynamics of the network using a frozen copy of the learned weight matrix where the off-diagonal structure was removed and apply plastic changes that result from the dynamics to the original weight matrix (red curve). We can see that in the former, the learned connectivity is reinforced and in the latter, the learned connectivity degrades. Off-diagonal weights (the y-axis) are quantified by averaging over the weights in the 80 by 80 blocks in the lower diagonal, for the 30 different clusters. The curves are the means over the 30 clusters and the error bars one standard deviation. (TIF) [file pcbi.1007606.s002.tif]

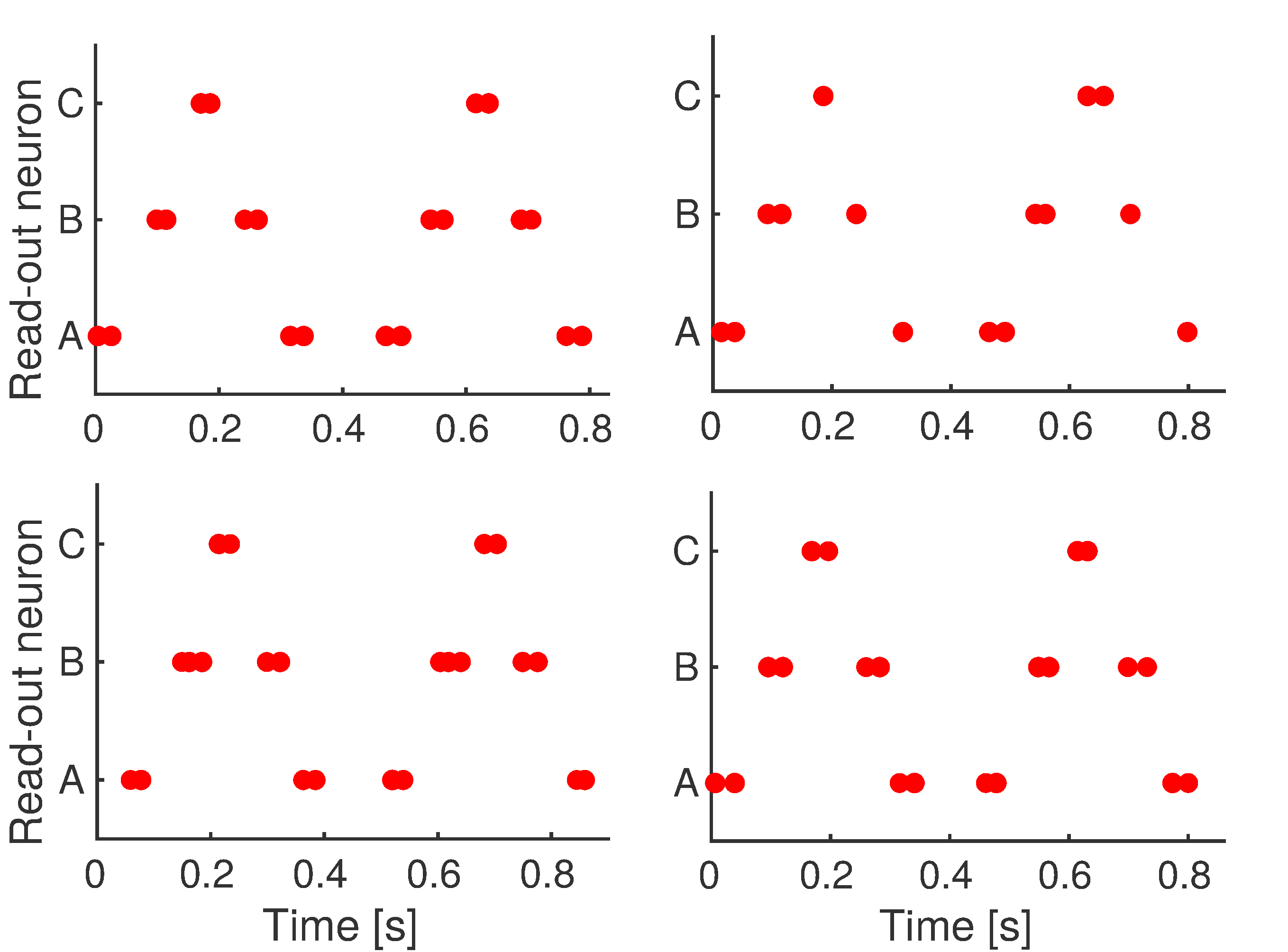

Supplement: S3 Fig — The sequence ABCBA is relearned four times for 12 seconds each. Before relearning, the read-out weight matrix WRE was always reset. When active, read-out neurons fire two spikes on average +/− one spike. This variability is a consequence of the noisy learning process. (TIF) [file pcbi.1007606.s003.tif]

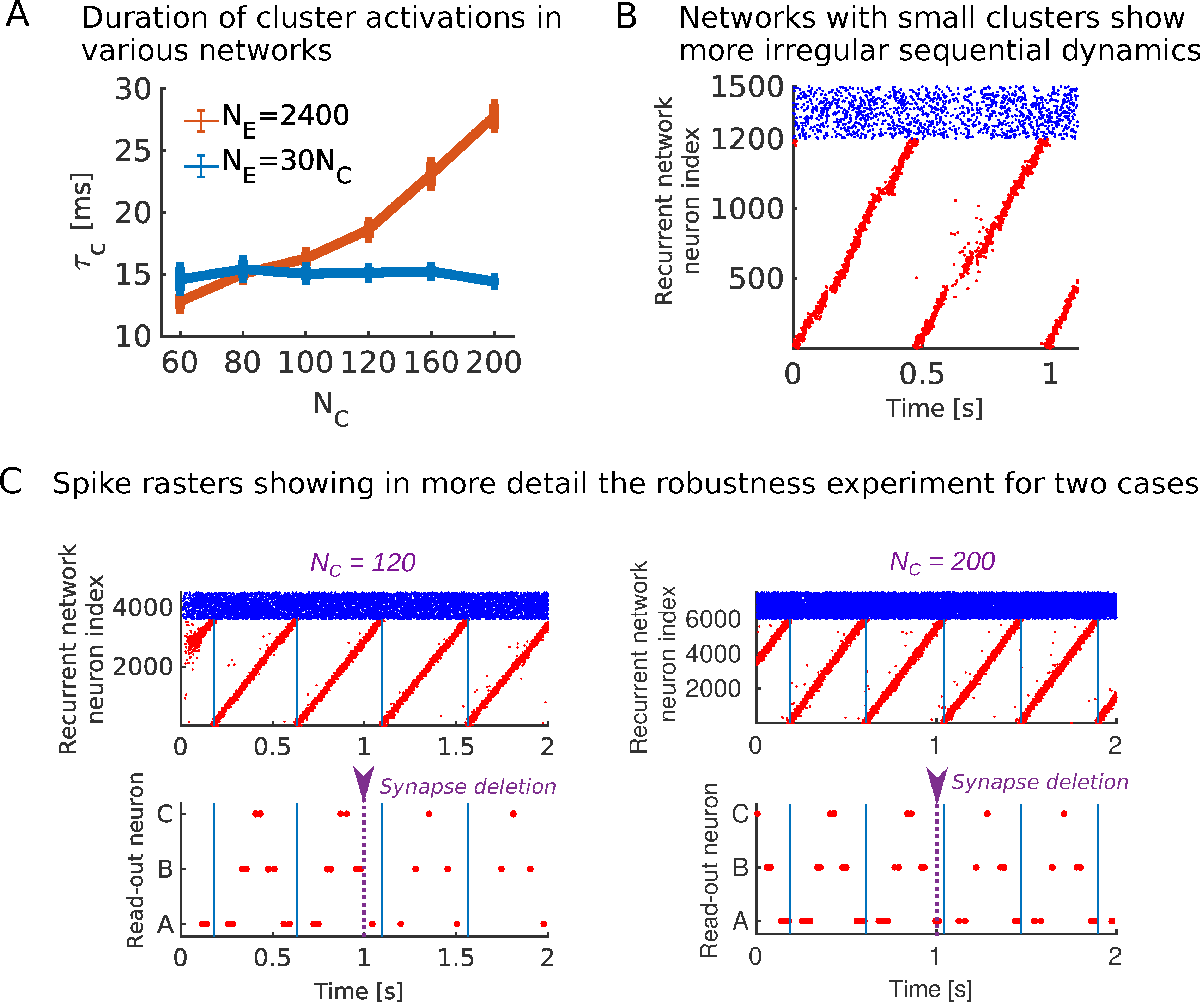

Supplement: S4 Fig — (A) Duration that a cluster is activated as a function of network size (B) Raster plot of sequential dynamics for NE = 1200 and NC = 40, after training. We observe that by reducing the cluster size, the irregularities in the sequential dynamics are increased (compare with Fig 2). (C) Two raster plots showing two different levels of robustness (summary plot in Fig 5C). In both cases, at t = 1s (purple arrow), 40 read-out synapses are deleted for each cluster. Left panel: NC = 120, each read-out neuron fires two spikes before deletion and one spike after deletion resulting in ∼ 50% performance. Right panel: NC = 200, each read-out neuron fires two spikes before deletion and one or two spikes after deletion resulting in a higher performance (∼ 80%). (TIF) [file pcbi.1007606.s004.tif]

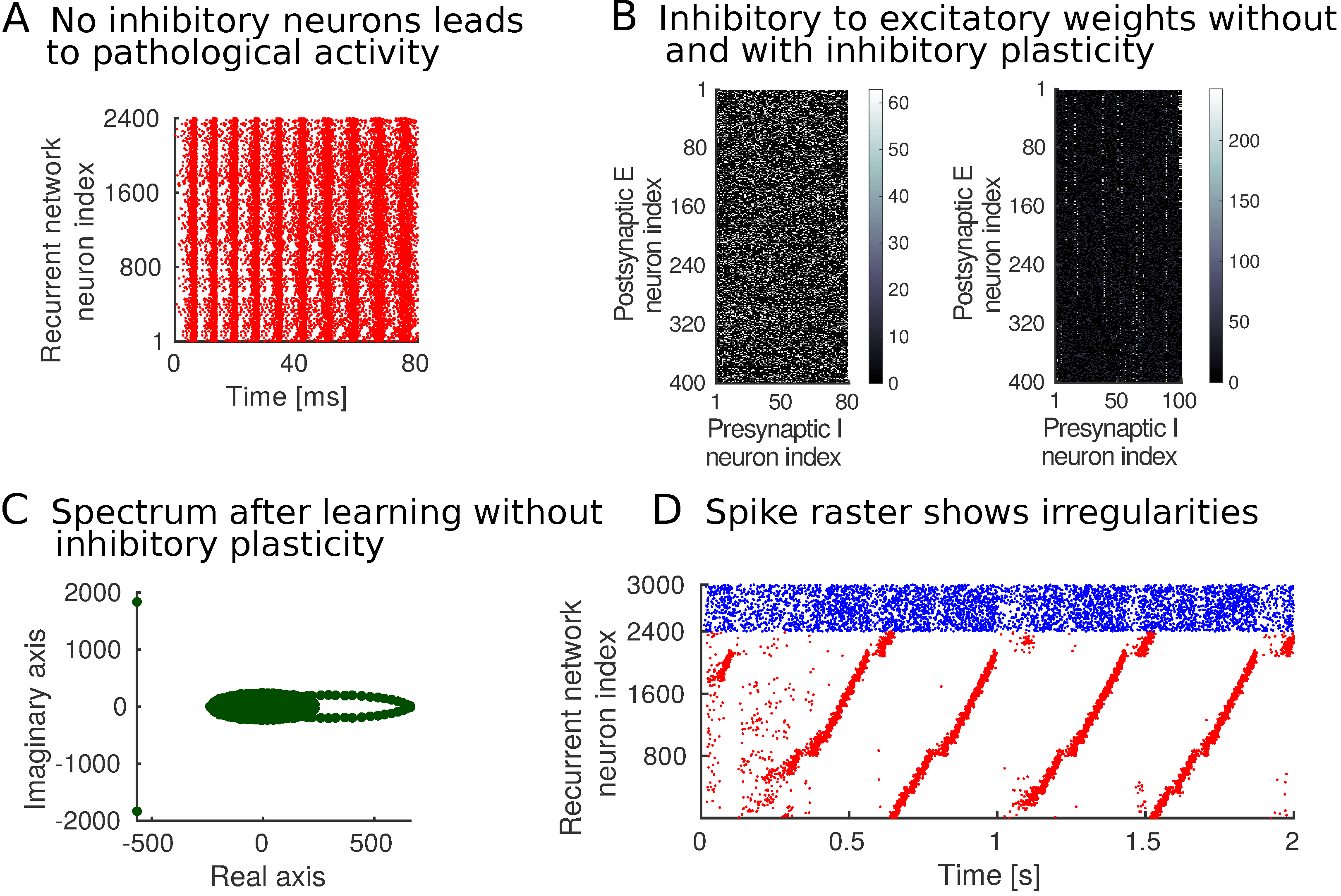

Supplement: S5 Fig — (A) Inhibitory neurons are necessary to prevent pathological excitatory activity. (B) The weights projecting from the inhibitory neurons to the excitatory neurons without inhibitory plasticity are random (left panel). The weights projecting from the inhibitory neurons to the excitatory neurons with inhibitory plasticity show some structure (right panel). (C) The full spectrum of the recurrent weight matrix after learning without inhibitory plasticity. (D) Without inhibitory plasticity, the sequential dynamics shows irregularities. The inhibitory plasticity allows for better parameters to be found to stabilize the sequential dynamics in the recurrent network. (TIF) [file pcbi.1007606.s005.tif]

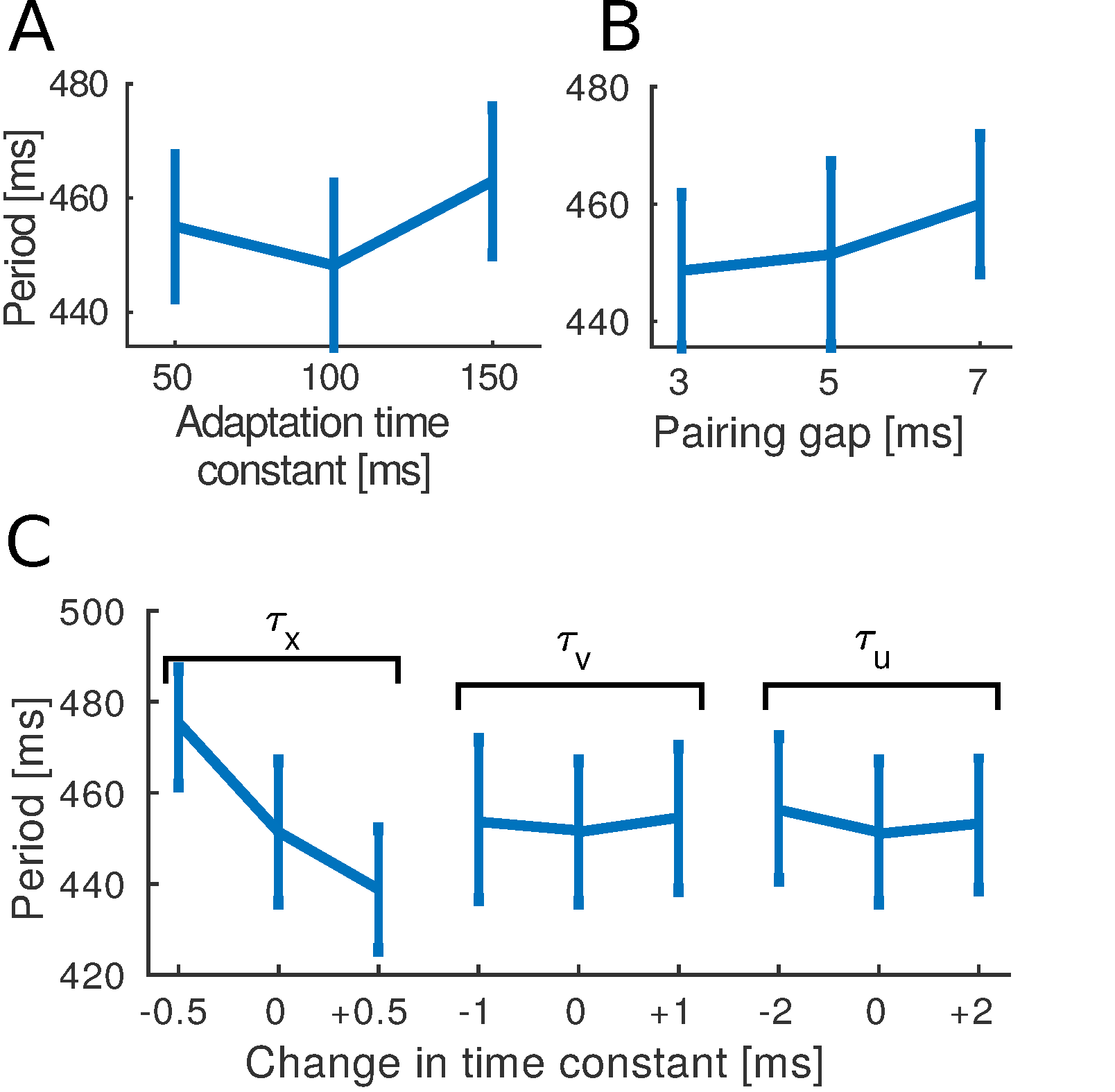

Supplement: S6 Fig — The periods of the sequential dynamics are computed after one hour of external stimulation and one hour of spontaneous dynamics. Only one parameter at a time is changed. (A) The adaptation time constant is varied. (B) The time gap between external sequential stimulations is varied. (C) The time constants of the voltage-based STDP rule are varied. The lines are guides to the eye and the error bars indicate one standard deviation. (TIF) [file pcbi.1007606.s006.tif]
