# Supplementary material for: Learning spatiotemporal signals using a recurrent spiking network that discretizes time
Source: PLoS Comput Biol. 2020 Jan 21;16(1):e1007606. doi: 10.1371/journal.pcbi.1007606 (PMC7028299; doi:10.1371/journal.pcbi.1007606)
Supplement: Attachment — Submitted filename: response_letter_final.pdf [file pcbi.1007606.s007.pdf]

Responses to the issues raised by the reviewers

Amadeus Maes, Mauricio Barahona, Claudia Clopath

November 2019

Reviewer 1

General comment

Your paper "Learning spatiotemporal signals using a recurrent spiking network that discretizes time" is an interesting and promising piece of work. You show that a simple architecture, consisting of a self-organizing recurrent network and an output layer, and trained through a form of 2-stage curriculum learning, can learn to reproduce complex temporal patterns that are much slower than the time scale at which individual neurons operate. This is a neat illustration of what a relatively simple model can do, and I believe it can become a foundation for a very interesting line of research!

The current version of the paper suffers from two weaknesses:

First, please revise your writing in several ways (see below for details)

Second, the paper introduces several special fine-tuned cases, and demonstrates that they work or don't work, but does not offer any conceptual sensitivity analysis. The paper will be stronger if you finish some (or ideally, all) of these lines of inquiry with proper sensitivity analyses. I recommend:

Major comments

1) Based on Fig2D, show how the quality of final stimulus replay (or any other measure of "success" for this model and this type of learning) depends on the exact pairing between the stimulus phase speed (100 ms) and the adaptation current time constant tau_a (100 ms). From Fig2D it seems that the stimulus is reproduced by the network so well largely because the neurons are "hard-tuned" to inactivate at exactly the speed you are using for stimulus transitions. It is not necessarily a problem, but it is an important aspect of conceptualizing the model. You could keep the stimulus fixed, change tau_a, and plot the replay quality (tau_a).

We thank the reviewer for this comment and address the point by doing a sensitivity analysis (see new supplementary figure on sensitivity analysis, Fig. S6). We find that the sequential dynamics itself is holding for a wide range of parameters. The mean period of the sequential dynamics in the recurrent network can vary slightly depending on exactly which parameters are changed (see Fig. S6 for more details).

2) For the comparison of large and small networks, make your statements more clear. The two plots shown in Figure 4A are practically identical, yet you make statements about these two models (large and small) both in the figure caption, and in the text. If there are differences there, in terms of time required for training, and/or their robustness, please show these numbers on the figure.

We agree that this was not clear and address this by removing the figure and replacing it with the new figure 5C. This figure shows how an increasing cluster size increases recall performance. The cluster sizes are shown in the legend of figure 5C. The training time is now more precisely stated in the text: *"The total learning time (Δ_t) is varied with the cluster size, $N_C\Delta_t$, because smaller clusters learn slower, since smaller clusters need larger read-out synaptic strengths to drive the same read-out neuron."*. In the caption we give a precise number: $\Delta_t = 960s/N_C$.

3) The most interesting analysis, in my opinion, would be one to explore the "optimal" number of clusters (and thus, the size of each cluster), for a model of N neurons, given a certain level of stimulus complexity, and a certain amount of noise (or neuron silencing). If for a given pair of (complexity, noise) you can -predict- the optimal network configuration, this would be a really interesting result, and it would make the paper more memorable, and impactful. Below I sketch how it can be done.

This is a very interesting proposal and not an easy question. To address this, we simulate networks with different cluster and network sizes. We summarize the results of these simulations in the new figure 5A. In the text we describe how the networks are scaled and how it affects the dynamics: *"We investigate several scaling properties of the network. We first assess how the sequential dynamics in the RNN depends on the cluster size by increasing the number of excitatory neurons in each cluster (N_C), preserving the ratio of excitatory to inhibitory neurons (N_E/N_I). To preserve the magnitude of the currents in the network, the sparseness of the connectivity (p) also varies with N_C such that pN_C is constant. The same training protocols are used for each*

network configuration as described in the Methods. In Fig. 5A, we show that for a fixed number of clusters C, the mean period of the sequential dynamics exhibited by the RNN is largely independent of cluster size N_C. If we fix the number of neurons in the RNN, and in this way change the number of clusters C, the mean period of the sequential dynamics decreases with increasing cluster size N_C. We conclude that the sequential dynamics is preserved over a wide range of network configurations. The time scales in the dynamics depend on the number of clusters and network size.". Taken together, the new figures Fig. 5A and Fig. 5C suggest ways to construct a network when a certain complexity and noise level is given. When the number of neurons in the recurrent network is held constant, larger cluster sizes lead to longer individual cluster activations (see also the new supplementary figure S5A). This means that the discretization of time becomes more coarse while the robustness increases. In the discussion we summarize: *"Taken together, our numerical simulations suggest ways to scale our network. An optimal network configuration can be chosen given the temporal length of the target sequence, and requirements on the temporal precision and robustness. For example, we have shown that a network with $N_E = 6400$ and $N_C = 200$ can be chosen for a 400 ms target sequence that can be learned fast with good temporal precision and a robust replay. If the network configuration and size are fixed, this severely constrains the sequences that can be learned and how they are replayed.".*

Detailed comments

Page 1

"slow-switching dynamics [Schaub et al., 2015]" - Please clarify

We add as clarification: *"In slow-switching dynamics, for instance, the wide range of time scales is produced by having stochastic transitions between clusters of neurons [Schaub et al., 2015].".*

Later in the same sentence: "and different types of sequential dynamics" - what are the options here? Can they be succinctly summarized?

We mention this in the context of spanning a wide range of time scales. One example of a model that has a wide range of time scales is the slow-switching dynamics mentioned in the previous comment. The second example we give is sequential dynamics. By 'different types of sequential dynamics' we mean that the dynamics can emerge in different ways. To remove this ambiguity, we explicitly state this and give two different examples: *"Another example is sequential dynamics, where longer time scales are obtained by clusters of neurons that activate each other in a sequence. This sequential dynamics can emerge by a specific connectivity in the excitatory neurons [Chenkov et al., 2017, Setareh et al., 2018] or in the inhibitory neurons [Billeh and Schaub, 2018].".*

"use non-local information either in space or in time" - explain these terms to non-computational readers

We add for clarification: *"use non-local information either in space or in time to update weights. Such information is not available to the synaptic connection, which only has access to the presynaptic and postsynaptic variables at the current time.".*

Page 2

"All synapses are plastic under typical Hebbian learning rules" - is STDP considered a typical Hebbian learning rule? Please be clear about whether you use a simple Hebbian potentiation, or a more fancy rule, such as STDP.

We thank the reviewer for raising this. We mean here Hebbian learning in the general sense, where potentiation and depression both depend on the product of activity in the presynaptic and postsynaptic neuron. STDP is a typical Hebbian learning rule for spiking neurons specifically. To remove this ambiguity, we add an adjective in the text: *"all synapses are plastic under typical spike-timing dependent Hebbian learning rules".*

"Thus, the recurrent network encodes time and the read-out neurons encode 'space'." - I am not sure I can understand this sentence. Could you please restate it, making it more clear?

We believe that the origin of the confusion might be the use of the word 'space'. A spatiotemporal signal has a temporal component and a 'spatial' component. In the introduction we give a general definition of what a spatiotemporal sequence is: *"Many tasks and behaviours in neuroscience consist of learning and producing flexible spatiotemporal sequences, e.g. a 2-dimensional pattern with time on the x-axis and any other observable on the y-axis which we denote here in general terms as the "spatial information".".* Later in the introduction we write: *"In this way, the recurrent network encodes time and the read-out neurons encode 'space'. As discussed above, we use the term 'space' to denote a temporally-dependent observable, be it spatial position, or phase, or a time-dependent frequency, or a more abstract state-space.".*

"Similar to the liquid state-machine" - remind the reader what a liquid state-machine is.

We remind the reader briefly by adding: *"Similar to the liquid state-machine where the activity in a recurrent network is linearly read-out by a set of neurons, we can learn different dynamics in parallel in different read-out populations".*

"Time is discretized in the recurrent spiking network by C clusters of excitatory neurons" 1) How time can be discretized by a cluster of neurons? Isn't time discretization a feature of your model, i.e. a step at which you perform your calculations? After re-reading the paper 2 times, I think I know what you mean, but I was very

lost the first time I read it. Please clarify. 2) mention what general type of a model you use for your neurons. (something like a "differential equation with 10 global parameters and 7 hidden variables per neuron, and a hard reset for spiking") If there a short reference for your model, cite it 3) introduce the concept of clusters separately. At this point I was really confused whether those 'clusters' you mentioned were just a metaphor, or whether they were a defining feature of your network. I also wondered whether they arose naturally, through the dynamics of activation, or whether they were introduced in the network deliberately.

We thank the reviewer for this comment, it was indeed confusing. The clusters (i.e. groups of neurons) are deliberately introduced by assuming that they share the same input. The discretization emerges after learning, as the C clusters are sequentially activated. To make the model more clear we divide the first section of results in three subsections: architecture, plasticity and learning scheme. The architecture subsection the concept of clusters and the neuron models: *"The recurrent network is organized in C clusters of excitatory neurons and a central cluster of inhibitory neurons. All excitatory neurons follow adaptive exponential integrate-and-fire dynamics [Brette and Gerstner, 2005] while all inhibitory neurons follow a leaky integrate-and-fire dynamics. The inhibitory neurons in the RNN prevent pathological dynamics. The aim of this module is to discretize time into C sequential intervals, associated with each of the C clusters. This is achieved by learning the weights of the recurrent network. The neurons in the excitatory clusters then drive read-out neurons through all-to-all feedforward connections. The read-out neurons are not interconnected. The target sequence is learned via the weights between the driver RNN and the read-out neurons."*. In the learning scheme subsection we explain the time discretization: *"During the first stage of learning, all neurons in each cluster receive the same input in a sequential manner. As a result of this learning stage, the recurrent spiking network displays a sequential dynamics of the C clusters of excitatory neurons. Neurons within each cluster spike over a time interval (while all neurons from other clusters are silent), with the activity switching clusters at points $t = [t_0, t_1, ..., t_C]$ so that cluster i is active during time interval $[t_{i-1}, t_i]$. Thus, time is effectively discretized in the RNN."*.

"This means that at each time only a subset of neurons spike, i.d. the neurons belonging to the same cluster." 1) What does "this" refer to? Please clarify. 2) Why does the previous sentence mean ("this means") that at each time only a subset of neurons spike? Why cannot all of them spike at once? 3) I don't know what "i.d." means, and I could not immediately google it, which may suggest that it is not a widely used abbreviation.

'i.d.' indeed does not exist, we made a typo. To clarify the other issues raised here, we write now: *"Neurons within each cluster spike over a time interval (while all neurons from other clusters are silent), with the activity switching clusters at points $t = [t_0, t_1, ..., t_C]$ so that cluster i is active during time interval $[t_{i-1}, t_i]$."*.

"At time t in $[t_{i-1}; t_i]$ the read-out neurons are solely driven by the neurons in cluster i, with some additional background noise. This discretization enables Hebbian plasticity to bind the read-out neurons to the neurons active in the relevant time-bin." - this part is really unclear. Please rewrite and clarify.

To clarify, we add an example and rewrite the text as follows: *"Neurons within each cluster spike over a time interval (while all neurons from other clusters are silent), with the activity switching clusters at points $t = [t_0, t_1, ..., t_C]$ so that cluster i is active during time interval $[t_{i-1}, t_i]$. Thus, time is effectively discretized in the RNN. During the second stage of learning, the read-out neurons receive input from a set of excitatory supervisor neurons. The discretization of time enables Hebbian plasticity to form strong connections from the neurons in the relevant time bin to the read-out neurons. For instance, if we want to learn a signal which is 'on' during $[t_{i-1}, t_i]$ and 'off' otherwise, a supervisor neuron can activate the read-out neuron during that time interval so that connections from cluster i to the read-out neuron are potentiated through activity (who fires together, wires together). This means that, after learning, the read-out neuron will be activated when cluster i is activated. In general, the read-out layer learns a multivariate signal of time, i.e., the neurons in the read-out layer encode the D different dimensions of the target signal: $t \to \phi(t) = [\phi_1(t), \phi_2(t), ..., \phi_D(t)]$."*.

"Here however, we use the voltage-based STDP plasticity rule at the excitatory to excitatory neurons" - is it true for all connections in the model, or only for some of them?

This is true for all the E-to-E connections. We add the word 'all' in the mentioned sentence.

"In the first stage, a feedforward weight structure is embedded into the recurrent network." - what does the word "embedded" mean in this context? Does it just mean that you initialize the network with some feed-forward way, or does it have a deeper meeting? While reading, at this point I assumed that you were describing some peculiar initial state, but without going to the "Methods" section, I was not sure whether my guess was correct. Please try to explain: 1) why you are trying to do something, 2) what is it exactly that you're trying to do, 3) how you're doing it. "The recurrent network is initialized as a balanced network with random connectivity." 1) You just said that you "embedded" some FF connectivity in it, and now you are saying the connectivity is random. At the first pass, it read as a contradiction. 2) Please clarify what "balanced" means in this context.

'Embedded' here is a synonym for 'learned'. We acknowledge the confusion raised by not properly defining the meaning of 'embedding'. To avoid confusion we remove the word 'embedded' and replace it with 'learned'. Additionally, we describe the initial weight matrix now more in detail, followed by a description of the initial dynamics: *"We give here further details of the first learning stage, where a recurrent network is trained to produce a sequential dynamics. To this end, we initialize the weight matrix so that each synaptic weight between*

two neurons is non-zero with probability p. The weights that are zero remain zero at all times, i.e. the topology is fixed. We set the initial values of the non-zero weights in the recurrent network such that the dynamics is irregular and asynchronous (i.e., a balanced network, see Methods for details).".

"To embed a feedforward structure, the network is stimulated in a sequential manner" - this return to a topic you abandoned 2 sentences ago makes it harder to stay on track.

This comment follows up on the previous comment. We want to show how to train a recurrent network such that it exhibits a sequential dynamics. We rewrite the section to describe all aspects in more detail. Six separate paragraphs describe the different aspects: (i) The initialization of the network; (ii) the sequential training protocol; (iii) stability of the learned connectivity structure; (iv & v) the link between the spectrum of the learned connectivity and the dynamics that emerges from it; (vi) mechanistic description of spontaneous dynamics.

"(rate of 18 kHz for 10 ms)." - you have not yet mentioned the time step of your calculations; it may be better to mention it at some point before this sentence, to put these numbers in perspective. But also, I am confused about this section, as in the very end of Methods section on page 14 you are saying that you worked with a time step of 0.1 ms. Would not 5 kHz be the highest possible frequency of spiking achievable in a model that is discretized at 10 kHz? How can you use 18 kHz then?

We thank the reviewer for pointing this out. Within the brackets we clarify: *"(rate of 18 kHz for 10 ms, assuming a large input population)"*. The training protocol in the methods also mentions: *"This high input rate does not originate from a single external neuron but rather assumes a large external input population.* Multiple spikes can arrive during every time step of simulation.".

"During the stimulation, neurons in the same cluster fire spikes together, which will strengthen the intra-cluster connections bidirectionally through the voltage-based STDP rule" 1) This sentence and 2-3 sentences before it are intriguing, but at this point the reader does not yet know -why- you do what you do. Is it the experiment already? Or are you trying to build a certain architecture using something like a curriculum learning, through inputs alone? Please make sure to describe what you are trying to achieve, and why you are trying to achieve it, before describing how you do it. 2) I always thought that the "canonical" STPD does not link neurons that spike together, but rather potentiates synapses connecting neurons that fire with a small delay of several ms. With 0.1 ms step and high-frequency stimulation, this difference could be very noticeable. Yet here you say that synchronous firing strengthened within-cluster connections bidirectionally. Is it because your STDP is symmetric? Or is it because neurons fire so much that they develop tight synfire chains within each cluster? Please clarify.

We thank the reviewer for this comment on the plasticity rule. The comment relates to an earlier comment on why we want to develop the structure. As stated in the paragraph on the model architecture and Fig. 1, we do not use the canonical STDP rule. Indeed, the voltage-based STDP rule we use allows the formation of bidirectional connections. This is what happens within the clusters: *"During the stimulation, neurons in the same cluster fire spikes together strengthening the intra-cluster connections bidirectionally through the voltage-based STDP rule [Clopath et al., 2010, Ko et al., 2013]".*

"When the time gap between sequential stimulations is increased during the training phase, there is no pre/post pairing between clusters anymore." - this sounds like the first true "Result", but I am still not sure what question you are trying to answer. Stating a question upfront would help a lot.

"This leads to slow-switching dynamics as opposed to sequential dynamics" - please clarify 1) what the word "this" refers to, and 2) what is the "slow-switching" dynamics, and how is it different from "sequential dynamics". You have a whole figure on it (Figure S1), so it would be really useful to define this term here, with a few references, and explain what this result means.

We address the two points by extending the explanation. The text reads now: *"If the time gap between sequential stimulations is increased during the training phase, so that the gap becomes too long with respect to the STDP time window, then there is no pre/post pairing between clusters and the ensuing dynamics loses its sequential nature and becomes a slow-switching dynamics [Litwin-Kumar and Doiron, 2014, Schaub et al., 2015] (Fig. S1). In slow-switching dynamics, clusters of neurons are active over long time scales, but both the length of activation and the switching between clusters is random. This is because the outgoing connections from cluster i to all other clusters are the same in a slow-switching network. To summarize, a connectivity structure emerges through biophysically plausible potentiation by sequentially stimulating the clusters of excitatory neurons in the recurrent network. When the gap between activation intervals is sufficiently small compared to the STDP window, the connectivity structure is such that intra-cluster weights and the weights from successive clusters* $i \to i+1$ *are strong.".*

"The connectivity pattern is therefore stable" - from the Abstract, I remember that overall you found this result intersting. If it is true, please expand your writing a bit, and explain why this is an interesting statement. Were you concerned that the dynamics would degrade? Are there previous studies that suggest the risk of degradation? If so, reference these studies, and explain your motivation, to properly frame this result.

We thank the reviewer for this important comment. We extend the stability analysis to frame the stability result better. In general, it is not trivial that a learned connectivity remains stable under spontaneous activity

[Morrison et al., 2007]. To show stability, we approach this in a way similar to the study of [Litwin-Kumar and Doiron, 2014]. We dedicate a new figure (Fig. S2B) and paragraph to the stability of the learned network connectivity: *"After the synaptic weights have converged, the external sequential input is shut-down and spontaneous dynamics is simulated so that external excitatory Poisson spike trains without spatial or temporal structure drive the RNN. Under such random drive, the sequence of clusters reactivates spontaneously and ensures that both the intra-cluster and the connections from cluster i to cluster $i+1$ remain strong. In general, the interaction between plasticity, connectivity and spontaneous dynamics can degrade the learned connectivity and lead to unstable dynamics [Morrison et al., 2007]. To test the stability of the learned connectivity, we track the changes in the off-diagonal weights (i.e. the connections from cluster i to cluster $i+1$). After the external sequential input is shut-down, we copy the weight matrix and freeze the weights of this copy. We run the dynamics of the recurrent network using the copied frozen weights and apply plastic changes to the original weight matrix. This means that we effectively decouple the plasticity from the dynamics. Indeed, when the dynamics is sequential, the off-diagonal structure is reinforced. When the off-diagonal structure is removed from the frozen copied weight matrix, the dynamics is not sequential anymore. In this case, the off-diagonal structure degrades. We conclude that the connectivity pattern is therefore stable under spontaneous dynamics (Fig. S2)."*.

"The feedforward weight embedding changes the spectrum of the recurrent weight matrix." - This sounds like a beginning of a new thought, which clearly deserves a new paragraph. I would also strongly suggest that you start this paragraph with 2-3 sentences, explaining what is a spectrum of a recurrent weight matrix, and how to interpret it (with 1-2 references). Make it easier for non-mathy (or intermediate-mathy) people to relate to your work.

Figure 2C (The drawing with an ellipse): While I know the idea of spectral analysis, I don't work with it daily, and with my level of background, this panel is completely impenetrable for me. I don't know how to read it, and what it means. I think the paper would much improve if this section about eigenvalues, and the Figure 2, are better referenced, and better explained.

"Analysis of a simplified linear rate model shows that the imaginary parts linearly depend on the strength of the feedforward embedding" - can you please expand on that, including why you did it, how you did it, and what was the result? Ideally, frame it as a sequence of simple statements. There's a known method. Here's how it works. In our case, conceptually, we could have expected to see this or that (which would have meant this or that). In practice, we observed this. Some aspect of it was surprising (what exactly), so we ran this additional model (why and how). We observed this. Here's what it means.

We thank the reviewer for raising this issue. We want to establish a link between the learned weights and the nonlinear spiking dynamics. This indeed deserves a new paragraph and we write to this end: *"We next studied how the spectrum of the recurrent weight matrix is linked to the sequential dynamics. In linear systems, the eigenvalues of the connectivity matrix determine the dynamics of the system. In a nonlinear spiking model, the relationship between connectivity and dynamics is less clear. The connectivity after learning can be seen as a low-dimensional perturbation of a random matrix. Such low-dimensional perturbations create outliers in the spectrum [Tao, 2013] and change the dynamics [Mastrogiuseppe and Ostojic, 2018]. Here, we have carried out a similar spectral analysis to that presented in [Schaub et al., 2015] (see Figs. 7 in the Methods and S2 in the Supplementary material). The weight matrix has most of its eigenvalues in a circle in the complex plane (Fig. 2C) with eigenvalues associated both with the balanced nature of the network, but, importantly, also with the sequential structure (Fig. 2C). As the temporal backbone develops through learning (as seen in Fig. S2). it establishes a spectral structure in which the pairs of leading eigenvalues with large real parts have almost constant imaginary parts."* The previous text introduces the goal of the analysis and frames figure 2C. We continue then the text by creating a link between the spectrum and the nonlinear dynamics: *"A simplified analysis of a reduced weight matrix (where nodes are associated with groups of neurons) shows that the imaginary parts of the dominant eigenvalues depend linearly on the strength of the weights from cluster i to cluster $i+1$ (see Methods, Fig. 7). Hence for this simplified linearised rate model, this results in an oscillatory dynamics where the imaginary part determines the frequency by which the pattern of activation returns due to the periodic excitation pattern. As shown in [Schaub et al., 2015], these properties of the linear system carry over to the nonlinear spiking model, i.e., the imaginary parts of the eigenvalues with large real parts determine the time scales of the sequential activity (Fig. S2)."*.

"A large adaptation current counteracts the recurrent reverberating activity to turn the activity reliably off." - but so, does it mean that the temporal dynamics or your spontaneous activity was not due to training, but due to this "hard-coded" adaptation in individual neurons? (see my next paragraph)

Page 4

"Therefore, as each cluster is spontaneously active in a sequence, that leads to a sequence length that reaches behavioural time scales" - So, are you saying that to jump from one cluster to another (say, from i to i+1), the activation in the cluster i should have reached a certain threshold, but at the same time the adaptation was driving this activation down? Does it mean that with adaptation too fast or too strong, there would be no propagation one cluster to another, while with adaptation too slow or too weak, clusters would not have "switched off"? But if so, would not your model work only if the adaptation is precisely tuned to the time

parameter of your stimulus? This may actually be my biggest question about this study, as whether this model generalizes well seems to hinge on it. If this architecture can work successfully only when adaptation at the level of individual cells is precisely tuned to the properties of the stimulus encoded by the entire network, this could be a strong limitation (in the spirit of non-local tuning). If however this architecture is robust, and can somehow tolerate a large range of adaptation properties, it would make it a good candidate for explaining encoding of temporal stimuli in the brain. The answer to this question is also tightly linked to some other questions raised in the paper: most importantly, the link between the number of clusters, the coding capacity of the network, and its robustness to noise (something I discuss more below). In order to relate to any analyses that play with the number of clusters in the network (in order to conceptually generalize them) the reader would need to know how these analyses would change if the adaptation time scale is not carefully pre-selected. If the quality of propagation is not too sensitive to the adaptation time, we need to know that. If, on the other extreme, the adaptation time sets the speed of this propagation, or needs to be strongly coordinated with some variables that set the speed of this propagation, we should know that as well, and keep it in mind, when interpreting any conceptual predictions from the model.

We thank the reviewer for this excellent question. The question is a more detailed version of the first major comment made earlier. It is true that a strong adaptation is important. However, as we show in our new simulations, the exact time constant is not important (Fig. S6). Additionally, we have shown that it is possible to scale the network and cluster size obtaining similar sequential dynamics (Fig. 5A). These results indicate that while adaptation needs to be strong, it does not need to be carefully tuned. As such, our results are robust to a wide range of parameters.

"simple yet non-markovian sequence" - please define what a markovian or non-markovian sequence means in this context, with a reference.

For clarity we keep this first sentence more general: *"After the sequential temporal backbone is learnt via the RNN, we can then learn a spatiotemporal sequence via the read-out neurons. To achieve this, during the second stage of training, the read-out neurons receive additional input from supervisor neurons and from interneurons (Fig. 3A). The supervisor neurons receive an external Poisson input with rate modulated by the target sequence to be learned (Fig. 3B). "*. We then continue with the explanation of what non-Markovianity means. It enters in the text: *"As a first example, consider a target sequence composed of states A, B, C activated in the following deterministic order: ABCBA. This is a non-Markovian state sequence because the transition from state B to the next state (A or C) requires knowledge about the previous state [Brea et al., 2013], a non trivial task that requires information to be stored about previous network states, potentially over long time periods."*. The cited paper studies sequence learning in a top down manner, where there are different requirements on the architecture depending on whether the sequence is Markovian or not.

"This task is non-trivial and it is unclear how to solve this problem in a generic recurrent spiking network with local plasticity rules." - this sentence would benefit from a bit of stylistic re-write, and also it clearly asks for a reference or two.

We rewrite this for clarity: *"... a non trivial task that requires information to be stored about previous network states, potentially over long time periods. Previous studies have proposed various solutions for this task [Maass and Markram, 2002 and Brea et al., 2013]. However, separating the problem of sequence learning in two stages solves this in a natural way."*.

"However, separating time and space solves this in a natural way." - very interesting, but also very unclear. It is the second time this theme is introduced, and I still don't understand it. If you feel strongly about it, please clarify what you mean, and try to explain to the reader why they should be excited about it (essentially, help them to understand how it may help them to conceptualize their own work later on).

The learning problem is separated in two stages. In the first stage, a temporal backbone is learned. In the second stage, read-out neurons, encoding the 'spatial' dimension of the spatiotemporal signal, are trained. In the introduction we clarify: *"Many tasks and behaviours in neuroscience consist of learning and producing flexible spatiotemporal sequences, e.g. a 2-dimensional pattern with time on the x-axis and any other observable on the y-axis which we denote here in general terms as the "spatial information"."*. The two stages of learning are detailed in the model architecture section of the results, with an example. We write here now: *"However, separating the problem of sequence learning in two stages solves this in a natural way."*.

"Our recurrent network (Fig. 2) is used here, where the first cluster is activated at time t." - This sentence would benefit from a rewrite.

Thank you for the comment. We rewrite the text in the following way: *"The recurrent network trained in the first stage (Fig. 2) is used to encode time. The underlying assumption is that a starting signal activates both the first cluster of the recurrent network and the external input to the supervisor neurons, which activate the read-out neurons."*.

"Due to the voltage-based plasticity, synapses are potentiated from neurons in the recurrent network and read-out neurons that fire at the same time" 1) I am not sure you mentioned before that these synapses are special. Please introduce it better. 2) Please explain how this special "voltage-based plasticity" helps here, compared to "more standard" Hebbian plasticity the reader may be thinking about.

We thank the reviewer for raising this point here. In the model architecture section (first section of the results), we now dedicate a subsection to this: *"In previous models, the learning schemes are typically not biologically plausible because the plasticity depends on non-local information. Here, however, we use the voltage-based STDP plasticity rule in all the connections between excitatory neurons (Fig. 1B)"*. This indicates that we use no different plasticity rule. In the learning scheme subsection we use a specific example to explain: *"For instance, if we want to learn a signal which is 'on' during $[t_{i-1}, t_i]$ and 'off' otherwise, a supervisor neuron can activate the read-out neuron during that time interval so that connections from cluster i to the read-out neuron are potentiated through activity (who fires together, wires together). This means that, after learning, the read-out neuron will be activated when cluster i is activated."*.

"To that end, we trained our recurrent network with clusters as small as one neuron." - Personally, I find this line of inquiry very interesting, as it speaks of brain architecture optimization, in terms of robustness to perturbation. However, you don't really explore this question, but look at one extreme case, and then make a prediction based on this extreme case: "In summary, the choice of cluster size is a trade-off between network size on the one hand and robustness on the other hand." I wonder whether a proper analysis of this relationship could be one of the main points of the paper, if run properly. What is the optimal number of clusters, for a stimulus of given complexity (say, a number of unique state transitions, or even just a number of states for a randomized sequence), and given amount of noise / perturbation?

This comment relates to the 3th major comment where the idea of brain architecture optimization is brought forward. We simulate a large number of networks with varying network and cluster sizes to better understand what is going on, and summarize the results in the new figures 5A, 5C and S5A.

Page 9

"These clusters are sequentially active by embedding a feedforward connectivity structure into the weights of the network." - the semantics is off here. If you think of it, clusters cannot be active by embedding. Please rewrite.

We rewrite this sentence as follows: *"The first module provides a temporal backbone that discretizes time, implemented by a recurrent network where excitatory neurons are trained into clusters that become sequentially active due to strong inter-cluster and cluster i to cluster $i+1$ weights."*.

"The time scales tau_c and tau_p are dependent on the cluster and network size, the average connection strengths within the clusters and adaptation. Smaller cluster sizes lead to a smaller tau_c and tau_p increases with network size when the cluster size is fixed." - arguably you don't address it in the paper, but only hint at it. To put it in a slightly exaggerated way, there is no summary plot among your figures that would have cluster size as an X axis, and some time-related measure as an Y axis. Or, time-related measure as X, encoding quality as Y, and several (more than 2) cluster sizes as different lines. If you don't think these summary analyses belong here, please restate your statements as hypotheses. If however you'd like to keep them as statements, please run appropriate analyses, and visualize them in one of the figures (I would definitely advocate for this second approach!)

This is an excellent point and relates again to the 3th major comment. We make these statements stronger by training networks with varying cluster and network sizes.

METHODS

Pages 10-11

"Membrane potential dynamics" - as I mentioned before, I am not quite sure how to classify your model, and this entire section does not contain any references. Your model seems to be simpler than HH, but more fancy than Izhikevich, and you are using a computationally expensive exp() function. The obvious question is: why? Would not integrate-and-fire be enough? Or a classic Izhikevich neuron? Are you using this model just because that was the first model you thought of, or do you NEED some of its properties to make your model work? I don't necessarily suggest that you run a simulation with different types of neurons, but if you know that you had to reach this level of complexity, perhaps because of some prior pilot experiments, I would really want you to write about that. And even if you use this model just because it was a reasonable "first choice", I would like to know about it, to avoid second-guessing.

We thank the reviewer for bringing this up. Earlier studies use this particular neuron model in combination with voltage-based STDP. However, the qualitative results should remain largely unchanged across different neuron models. Indeed, initial pilot experiments with classical leaky integrate-and-fire dynamics for all neurons suggested that at least the second stage of learning worked as well. We add at the end of the first paragraph of the methods section: *"The dynamics was chosen based on previous models, with parameters for the dynamics and plasticity to a large extent conserved [Litwin-Kumar and Doiron, 2014]. More simple integrate-and-fire dynamics should lead to the same qualitative results, given that the parameters are appropriately changed (data not shown)."* At the beginning of the paragraph the reference for the model was added: [Brette and Gerstner, 2005].

To rephrase, I would be grateful if you put your model in a context (with some references), and commented on your choice for the level of complexity in modeling your neurons. A few choices that jump at me as I read these section are your attention to refractory periods and adaptation currents. Would it be fair to say that

these are important for your model? Can you explain (or at least speculate), how and why? Any background for these choices would be very helpful.

The refractory period is not particularly important and only defines an upper limit for the rate of a neuron. The adaptation is more important. We have a high adaptation in the recurrent network and a low adaptation in the read-out neurons. We want neurons in the recurrent network to be very sparsely active and the read-out neurons should on the other hand be able to fire at a wide range of rates. To clarify, we add in the paragraph: *"There are two different regimes, one for each part of the model. Excitatory neurons in the recurrent network have a high adaptation current while excitatory neurons in the read-out network have no adaptation. This is to allow for a wide range of firing rates in the read-out network, while spiking is more restricted in the recurrent network. Differences in the refractory period are there for the same reason, but are not crucial."*

Similarly, where you use different models for different neurons ("only non-zero during learnig" etc.), it would be nice to get an idea for your rationale. Why was it helpful to make different neurons different?

The supervisor- and inter-neurons in the read-out network are there for training the read-out synapses. We clarify by adding: *"To help learning, there are two additional types of neurons in the read-out network."* More in detail we add below the equation for the read-out neurons: *"g^{RS} is the excitatory input from the supervisor neuron (supervisor input only non-zero during learning, when the target sequence is repeatedly presented). g^{RH} is the inhibitory input from the interneuron (only non-zero during learning, to have a gradual learning in the read-out synapses)."*

Page 12

"Plasticity": my general feedback for this statement is similar to that for membrane potential dynamics: can you please use several sentences to hint why you settled on this level of complexity for the dW/dt equation, and how you have arrived at your constant? Not in deep detail, but conceptually? What were your criteria? Say, for each neuron you use 5 different proxies for its activity: V, s, u, v, and x. Why do you need all five? Why 3 (V, s, filtered-s) would not be enough? Similarly, why u and v use two just slightly different taus? Is it to create an asymmetric waveform over time, or is it just a result of manual tweaking? I realize that it would be counter-productive to "justify" any of these micro-choices, but it would be very useful to have some basic understanding of what you were trying to achieve, and how you figured whether you achieved it.

We thank the reviewer and address the point in two ways. Firstly, we need a plasticity rule with a rate-dependency, the voltage-based STDP rule has this feature [Clopath et al., 2010]. This becomes especially clear in the read-out synapses. We give an example in the model architecture section: *"During the second stage of learning, the read-out neurons receive input from a set of excitatory supervisor neurons. The discretization of time enables Hebbian plasticity to form strong connections from the neurons in the relevant time bin to the read-out neurons. For instance, if we want to learn a signal which is 'on' during $[t_{i-1}, t_i]$ and 'off' otherwise, a supervisor neuron can activate the read-out neuron during that time interval so that connections from cluster i to the read-out neuron are potentiated through activity (who fires together, wires together). This means that, after learning, the read-out neuron will be activated when cluster i is activated."*. Secondly, the new sensitivity analysis (Fig. S6) shows that the sequential dynamics is robust for a wide range of plasticity time constants.

"Heaviside function" - please remind the reader the definition of this function, in brackets

A mistake was made here, we correct: *as $R(.)$ denotes the linear-rectifying function ($R(x) = 0$ if $x < 0$ and else $R(x) = x$).*

Reviewer 2

General comment

I believe this type of model approach is quite important and helpful to provide a better understanding of underlying principles in experimental observations. While interesting and potentially relevant for the study of the gap between behavioral and neural timescales, a substantial portion of the model designs and simulation analyses need to be presented with much better explanation of "meaning", to clarify the rationale behind the model choice and the goal of each test. A major issue is that it is not clear what exactly can be learned from these results, what kind of problem can be solved with the current model (but cannot be solved by previous ones), and to which experimental observations each simulation result is relevant. I believe that some major issues, including the above, need to be addressed before publication can be recommended.

We thank the reviewer. To address the issue raised here, we have rewritten the paper extensively. We emphasize now the rationale behind the model and analyses. In the responses below, we give detailed information on how the writing and analyses are extended.

Major comments

1. Authors need to discuss specifically what is the difference between the current model and the previous ones. For example, learning of spatio-temporal sequence with RNN has already been implemented in previous studies.

For example, what is the difference and advantage of the current model compared with the model in Chenkov, Sprekeler & Kempter, PloS Comput Biol 2017? If authors think that the current model uses more biologically plausible components, as mentioned in abstract "… current computational models do not typically use realistic biologically plausible learning.", there are some model studies that use realistic, biologically plausible learning rules. For example, see Park, Choi & Paik, Scientific Reports (2017) and also Lee et al. bioRxiv 525220 (2019). In addition, there are a number of successful models for the spike sequence learning (e.g. tempotron by Sompolinsky). Thus it will be helpful that authors discuss these previous approaches and compare them with the current model, to clarify what kind of novelty the current model has.

We thank the reviewer for raising this issue. The work of Park, Lee, Choi and Paik, and the tempotron, are in a sense complementary to our work. We describe this as follows: *"Other studies have focused on classification of spatiotemporal signals. The tempotron classifies a spatiotemporal pattern by either producing a spike or not [Gütig and Sompolinsky, 2006]. More recent studies on sequential working memory propose similar model architectures that enable the use of Hebbian plasticity [Manohar et al., 2019]. For example, spatiotemporal input patterns can be encoded in a set of feedforward synapses using STDP-type rules [Park et al., 2017, Lee et al., 2019]. Combining these approaches with our model might be an interesting line of future research."*. Second, a new paragraph discusses the learning rules used: *"In this paper, we use local Hebbian learning to produce a sequential dynamics in the recurrent network. This is in contrast with previous studies, where often a recursive least squares method is used to train the weights of the recurrent network [Rajan et al., 2016 and Hardy and Buonomano, 2018]. Hardcoding a weight structure into the recurrent network has been shown to result in a similar sequential dynamics [Chenkov et al., 2017 and Setareh et al., 2018]."* Then we continue by stating: *"Studies that do incorporate realistic plasticity rules are mostly limited to purely feedforward synfire chains [Fiete et al., 2010, Waddington et al., 2012, Zheng and Triesch, 2014]. Those studies focus on the generation of a dynamics that is sequential. In this regard, the contribution of our work is to use the sequential dynamics as a key element to learning spatiotemporal spiking patterns."*.

2. How can you control the timescale of temporal backbone in the RNN? Or is there any critical factor that can control it? It needs to be explained how this timescale changes in the model and how it is relevant to real biological mechanism to handle different behavioral timescales across tasks. In the current model, conversion from the neural timescale (ms) to behavioral timescale (hundred ms) seems to strongly depend on the transmission delay. How and how much the timescale can vary in the model? Can you explain how to make much longer backbones than the current ones? Also what happens if the supervisor signal length varies?

We thank the reviewer for this comment and extend our analysis. To answer this question, we train networks with varying cluster and network sizes. The effect on the time scales of the temporal backbone can be seen in the new figures Fig. 5A and Fig. S5A. However, once this temporal backbone is trained, the time scales are mostly fixed. A small modulation of the period ($\sim 10\%$) is still possible by changing the external input as we show in the new figure Fig. 5B. We add a paragraph to discuss this modulation: *"Another way to modulate the period of the sequential dynamics is to change the unstructured Poisson input to the RNN during spontaneous dynamics (i.e., after the first stage of learning). When the rate of the external excitatory input is increased/decreased, the mean period of the sequential dynamics in the RNN decreases/increases (Fig. 5B). These results suggest that the network could learn even if the supervisor signal changes in length at each presentation, assuming that both the supervisor and external Poisson input are modulated by the same mechanism. "*

3. In the intro, authors mention that "… the recurrent network encodes time and the read-out neurons encode 'space'. 'Space' can mean spatial position, but it can also mean frequency or a more abstract state space". I think the word "space" here is confusing, or not the best choice. Authors mentioned (in discussion) that "a set of read-out neurons that encode multiple dimensions of a signal, which we also call space". Does it mean the spatial structure of feedforward connectivity, or structure of input pattern (whatever the contents are..)? I understand that authors intended to express a generalized "spatio-temporal" information, but this needs to be better described to avoid confusion.

We thank the reviewer and agree that the word 'space' is confusing. Indeed, we mention 'space' at several points in the text without properly defining the meaning. We address this by first defining 'spatiotemporal sequence' in the introduction: *"Many tasks and behaviours in neuroscience consist of learning and producing flexible spatiotemporal sequences, e.g. a 2-dimensional pattern with time on the x-axis and any other observable on the y-axis which we denote here in general terms as the "spatial information"."* Later in the introduction we write: *"In this way, the recurrent network encodes time and the read-out neurons encode 'space'. As discussed above, we use the term 'space' to denote a temporally-dependent observable, be it spatial position, or phase, or a time-dependent frequency, or a more abstract state-space."*

4. How much information can be learned in the current network? i.e. what is the capacity of the current model network? Is it possible to train more than one non-Markov pattern in the same network? Is it biologically plausible, if your model uses a number of neurons only to learn one or just a couple of patterns?

We thank the reviewer for this very important question. To address this properly we add a new paragraph and a new figure (Fig. 4) to accompany this paragraph: *"We next wondered how multiple spatiotemporal signals can be learned. We hypothesized that, once the temporal backbone is established, multiple spatiotemporal sequences*

can easily be learned in parallel. As an example, we learn two sequences: ABCBA and DEDED. Here, D and E denote two additional read-out neurons (Fig. 4A). We assume that the model observes each sequence alternately for 2 *seconds at a time (Fig. 4B), although in principle it could also been shown simultaneously. After learning, the target sequences are encoded in the read-out weight matrix (Fig. 4C). In a regime of spontaneous dynamics the learned sequences can be replayed (Fig. 4D). We conclude that multiple sequences can be learned in parallel. Each separate sequence requires a separate set of read-out neurons. As such, the number of read-out neurons required increases linearly with the number of target sequences. "*

5. What are the roles of inhibitory neurons and inhibitory plasticity? Is there any particular condition required for them, except for preventing runaways dynamics? A more detailed role of inhibitory population might be important in the model.

This comment raises an important question. We address the question by simulating the network with and without inhibitory neurons, and with and without inhibitory plasticity. The findings are summarized in the new supplementary figure S6. Summarizing here, we can say that inhibitory neurons are necessary to avoid pathological seizure like activity. The role of inhibitory plasticity is more complex and detailed. When we look at raster plots of the dynamics after training without inhibitory plasticity (Fig. S6D), we see very irregular sequences. We add to the Methods section the following: *"Here, it allows to automatically find good parameters (see also Fig. S6)".*

6. Can you try any test to show the robustness of the model? For example, how many synapses can be destroyed without losing the trained information? I guess destroying RNN-readout connections might be critical. Then, is there any evidence that this system is relatively robust than others?

We thank the reviewer for raising this issue and we extend the robustness analysis accordingly. Read-out connections are destroyed for different networks in the new figure Fig. 5C (specific spike rasters shown in the new figure Fig. S5C). We then discuss the robustness of the sequential dynamics itself (Fig. 5D and Fig. S5B) where it is seen that the sequential dynamics produced by networks with very small clusters is not robust. A comparison of the robustness of this model with other models is hard to make. The architecture and number of neurons varies amongst many other parameters across different model studies. We add in the discussion: *"The robustness is a consequence of the clustered organization of the recurrent network. Previously proposed models are also robust to similar levels of noise [Nicola and Clopath, 2017, Nicola and Clopath, 2019]. While an exact comparison is hard to draw, we have shown that it is possible to retain robustness while moving towards a more biological learning rule."*
