# Supplementary material for: Learning spatiotemporal signals using a recurrent spiking network that discretizes time
Source: PLoS Comput Biol. 2020 Jan 21;16(1):e1007606. doi: 10.1371/journal.pcbi.1007606 (PMC7028299; doi:10.1371/journal.pcbi.1007606)
Supplement: Attachment — Submitted filename: Response letter.pdf [file pcbi.1007606.s008.pdf]

Responses to the remaining issues raised by the reviewers

Amadeus Maes, Mauricio Barahona, Claudia Clopath

December 2019

Reviewer 1

Overall, I am very happy with the edits, and I think the paper has become so much better with these additional analyses, and constructive rewrites! It is a pleasure to read; it provides a decent amount of background information, and answers most of the questions the readers might have.

My only concern is about the following two statements:

p 123: We set the initial values of the non-zero weights in the recurrent network such that the dynamics is irregular and asynchronous (i.e., a balanced network, see Methods for details).

p 408: The weights are initialized such that the network is balanced, i.e. it exhibits irregular asynchronous spiking dynamics.

As you can see, in the results you refer the reader to the Methods, but in the Methods, you essentially reiterate the same statement, and leave it at that. You don't describe why this network would be called "balanced" (I assume you could use a reference for that, as probably somebody used this word for chaotic before), you don't describe the criteria for "irregularity" (did you just observe it by eye, or have you automated it?), and you don't describe the actual process of "ensuring that it is balanced" (I'm guessing you just generated a network, tested it, and if it was bad, generated a different one - but it would be nice to state it). I would recommend that you add a sentence or two about that in the methods section (immediately following line 408).

Other than that, I'm happy with the paper, and recommend it for publication. Nice work!!

We thank the reviewer for pointing out this remaining issue.
We now provide additional information in the Methods about the meaning of a 'balanced network' and the specific construction we follow. Specifically, our setup involves scaling the parameters from the previous study by Litwin-Kumar and Doiron, and verifying that the scaled parameters indeed lead to irregular dynamics. We have explained this in Methods with the corresponding references:
"Our network is balanced in terms of inhibition and excitation, so that it displays irregular and asynchronous spiking. This is signalled by the coefficient of variation (CV) of the inter-spike intervals of the neurons being CV ~ 1, thus indicating Poisson-like spiking [Brunel, 2000]. In our construction, we initialise the weights of the network near the balanced state by scaling the weights of the balanced RNN in [Litwin-Kumar and Doiron, 2014] by the square root of the relative network size. We then verify that the scaled parameters indeed lead to irregular dynamics. The spiking dynamics is slightly more regular on average, with a mean CV ~ 0.8 for excitatory neurons and a mean CV ~ 0.9 for inhibitory neurons."

Reviewer 2

The authors have made a comprehensive and detailed revisions and addressed most of issues I raised. The revised manuscript is improved in some respects, particularly for the description of the robustness of the model and the modulation of the period of sequential dynamics. I have several questions and suggestions left, related to the capacity and of the model and its biological plausibility.

We thank the reviewer for their detailed feedback. We answer the remaining questions below and have incorporated the suggestions in the text.

1. The authors show that the network can learn multiple patterns (Fig. 4). However, this model seems to require a number of readouts, which I don't believe the optimal design of memory. For example, to memorize N patterns of the length L, how many readout neurons do you need? Can your model share the same readouts for pattern A and B? If not, is this biologically plausible model?

As shown in Figure 4, we need N read-out neurons to memorize N patterns. We have clarified this in the text:
"Each sequence requires a separate set of read-out neurons. Hence, the number of read-out neurons increases linearly with the number of target sequences.".
It is an open question how to increase the capacity of the model. We suggest a hierarchical organization as one possible mechanism. In the discussion, we have now added a sentence expanding this point:

"A hierarchical organization might also improve the capacity of the network. In our proposed serial organization, the number of spatiotemporal patterns that can be stored is equal to the number of read-out neurons. A hierarchical system could be one way to extract general patterns and reduce the number of necessary read-out neurons.".

2. Because the network uses a voltage-based STDP, the pattern needs to be trained multiple times in general (i.e. There is no one-shot learning mechanism in this model). Can you implement, or discuss any possible mechanism that address this issue? For example, synchronization between the temporal backbone and supervisor signal can make any changes of learning rate or efficiency?

We thank the reviewer for raising this point. In its current form, as the reviewer points out, we present the pattern multiple times and there is no one-shot learning. We have added a few lines expanding on how the framework could potentially be extended to one-shot learning. In the Discussion, we have added the following: *"In its current form , the target pattern needs to be presented repeatedly to the network and does not support one-shot learning through a single supervisor presentation. Although increasing the cluster size N_C can reduce the number of presentations, the size of the clusters would need to be impractically large for one-shot learning. Alternatively, increasing the learning rate of the plastic read-out weights could be a way to reduce the number of target presentations to just one [Nicola and Clopath, 2019]. However, it is unclear at present whether such high learning rates are supported by experiments [Sjöström et al., 2001]."*.

3. In the current model, the RNN module that encodes time information and the readout module for spatial information are separated. Do you have a good explanation that this is biologically realistic design, or can you suggest any possible experiment for finding evidences of this structure in biological brain? Then, what do you think is the advantage of separated circuits for spatial and temporal information? What happens when the network learns a spatio-temporally correlated information?

We thank the reviewer for this important point. To address this, we list some experimental findings in the discussion and discuss them in relation with the separation of time and 'spatial' information. We have added the following text:
"The ubiquity of sequential dynamics in various brain regions [Ikegaya et al., 2004, Jin et al., 2009, Mackevicius et al., 2019] and the architecture of the songbird system [Fee and Scharff, 2010] was an inspiration for the proposed separation of temporal and spatial information in our setup. As we have shown, this separation enables the use of a local Hebbian plasticity rule in the read-out synapses. Our model would therefore predict that perturbing the sequential activity should lead to learning impairments. Further perturbation experiments can test this idea and shed light on the mechanisms of sequential learning [Hemberger et al., 2019].".

4. Still I find the current model is very similar with previous models in some sense, except for some biological components. Can you better clarify the need of current model, with examples of tasks that cannot be performed with previous designs?

Previous designs that perform well typically use mathematical optimization, e.g., FORCE training [Sussillo and Abbott, 2009, Laje and Buonomano, 2013, Nicola and Clopath, 2017, Nicola and Clopath, 2019] or back-propagation through time [Werbos, 1990] to find the weights; hence the fundamentals of the learning process in such models are not biologically plausible. Although our model does not outperform those computationally-optimised approaches, we show that we can retain much of the performance and robustness while simultaneously moving towards a more biologically realistic model. At the same time, previous designs that use biological realistic dynamics [Chenkov et al., 2017, Setareh et al., 2018, Spreizer et al., 2019] and plasticity [Fiete et al., 2010, Waddington et al., 2012, Zheng and Triesch, 2014] have not moved beyond the production of simple sequential dynamics. In this context, our model be seen as an attempt to bring the current state of the art in local learning based on spiking networks closer to machine learning performance. We have added a qualification to the following sentence in the final paragraph of the Discussion:
"In summary, we have demonstrated that a clustered network organization can be a powerful substrate for learning, moving biological learning systems closer to machine learning performance. Specifically, the model dissociates temporal and spatial information and therefore can make use of Hebbian learning to learn spatiotemporal sequences over behavioural time scales.".
